# Myoelectric control algorithm for robot-assisted therapy: a hardware-in-the-loop simulation study

**DOI:** 10.1186/s12938-018-0622-1

**Published:** 2019-01-03

**Authors:** Juan C. Yepes, Mario A. Portela, Álvaro J. Saldarriaga, Vera Z. Pérez, Manuel J. Betancur

**Affiliations:** 1 Grupo de Automática y Diseño A+D, Cir. 1 #73-76, B22, Medellín, 050031 Colombia; 2 Grupo de Investigaciones en Bioingeniería, Cir. 1 #73-76, B22, Medellín, 050031 Colombia; 3 Facultad de Ingeniería Eléctrica y Electrónica, Cir. 1 #70-01, B11, Medellín, 050031 Colombia

**Keywords:** Assistive robotics, Electromyography (EMG) control, Powered exoskeletons, Biomedical signal analysis, Myoelectric control

## Abstract

**Background:**

A direct blow to the knee is one way to injure the anterior cruciate ligament (ACL), e.g., during a football or traffic accident. Robot-assisted therapy (RAT) rehabilitation, simulating regular walking, improves walking and balance abilities, and extensor strength after ACL reconstruction. However, there is a need to perform RAT during other phases of ACL injury rehabilitation before attempting an advanced exercise such as walking. This paper aims to propose a myoelectric control (MEC) algorithm for a robot-assisted rehabilitation system, “Nukawa”, to assist knee movement during these types of exercises, i.e., such as in active-assisted extension exercises.

**Methods:**

Surface electromyography (sEMG) signal processing algorithm was developed to detect the motion intention of the knee joint. The sEMG signal processing algorithm and the movement control algorithm, reported by the authors in a previous publication, were joined together as a hardware-in-the-loop simulation to create and test the MEC algorithm, instead of using the actual robot.

**Experiments and results:**

An experimental protocol was conducted with 17 healthy subjects to acquire sEMG signals and their lower limb kinematics during 12 ACL rehabilitation exercises. The proposed motion intention algorithm detected the orientation of the intention 100% of the times for the extension and flexion exercises. Also, it detected in 94% and 59% of the cases the intensity of the movement intention in a comparable way to the maximum voluntary contraction (MVC) during extension exercises and flexion exercises, respectively. The maximum position mean absolute error was $0.1^{\circ }$, $6.3^{\circ }$, and $0.3^{\circ }$ for the hip, knee, and ankle joints, respectively.

**Conclusions:**

The MEC algorithm detected the intensity of the movement intention, approximately, in a comparable way to the MVC and the orientation. Moreover, it requires no prior training or additional torque sensors. Also, it controls the speed of the knee joint of Nukawa to assist the knee movement, i.e., such as in active-assisted extension exercises.

## Background

The knee is the largest and most complex joint in the human body, and it depends on four primary ligaments, tendons, muscles and secondary ligaments to maintain its correct function. One of the main ligaments is the anterior cruciate ligament (ACL). The ACL is one of the most commonly injured ligaments in the knee. A direct blow to the knee is one way to harm the ACL, e.g., during a football or traffic accident. Nevertheless, most ACL injuries occur even without any contact with an object [1].

There are many traditional methods and devices to assist treatment. The study of new, applied technologies in areas such as Bioengineering and Automation has brought research and technology in robotic platforms that replace, enhance or rehabilitate lower limb disabilities. Within these applications, robotic systems have become a benefit for the rehabilitation of lower limb pathologies [2]. These studies have focused on the development of active orthosis, also defined as exoskeletons [3–6].

For example, in the case of ACL injuries, Hu et al. [7] reported in 2016 a robot-assisted therapy (RAT) rehabilitation investigation, simulating regular walking to examine the effects of long-term interventions using RAT rehabilitation on functional activity levels after ACL reconstruction. The study reported that the RAT treatment improved extensor strength and walking and balance abilities.

However, ACL injuries require various rehabilitation phases with the purpose of controlling pain and swelling, restoring pain-free range of motion (ROM), improving flexibility, normalizing gait mechanics, and establishing good quadriceps activation [8]. There are several international protocols for ACL injury rehabilitation such as the Accelerated ACL Reconstruction Rehabilitation Program of the Chester Knee Clinic & Cartilage Repair Center [9, 10], the Classic 1981 Protocol by Lonnie et al. [11], the ACL Reconstruction Rehabilitation Protocol of the Steadman Clinic [12], among others. These protocols report several rehabilitation phases for ACL injuries.

For the protocols mentioned above, there is the need to perform RAT rehabilitation of ACL injuries during other phases of the rehabilitation process before attempting an advanced exercise such as walking, e.g., when the patient is unable to execute a knee movement, due to the pain caused by the injury. In this rehabilitation phase, an active-assisted rehabilitation exercise may be conducted. During these types of activities, an external force provides assistance, mechanical or manual, since the muscle requires support to complete the movement [13, 14]. Moreover, during the traditional rehabilitation process of ACL injuries, the protocol uses knee active-assisted extension exercises. The subject uses the opposite leg to restore ROM during these exercises, e.g., and the healthy leg straightens the non-healthy knee from a $90^{\circ }$ flexion to $0^{\circ }$ [15].

Therefore, the specific problem addressed in this paper is to detect the motion intention and control a robotic rehabilitation system to assist the knee movement, i.e., such as in active-assisted extension exercises, but using an exoskeleton.

In order to detect the motion intention of a limb or joint, electromyography (EMG) signals have been used, and with this information, it has been possible to control rehabilitation systems [16]. There are many studies reporting surface electromyography (sEMG) signal processing algorithms to detect the motion intention of a limb or joint. Several studies reported algorithms that were implemented and tested offline [17, 18], and other algorithms were implemented online [19–22]. Also, some of them are currently under investigation [17, 18, 20–23] and others in the commercial stage, e.g., the algorithm reported by Hayashi et al. [19]. Also, several algorithms were tested in the knee joint [19] and other algorithms detect the motion intention in other joints [17, 18, 20–23]. Other studies were conducted with 1 [22], 2 [24], 3 [23], 4 [18], 10 [21], 12 [17], and 18 [20] healthy subjects. In the literature, there are sEMG signal processing algorithms that detect the motion intention. For example, myoelectric activity and a linear combination (*LC*) [19], feature extraction and a linear state-space model, autoregressive output structure with exogenous input (ARX) model, with multi-input single output [24]. Moreover, there exist EMG features and low-pass filter [20], Kalman filters [17], root mean square (RMS) envelope and a three-layer back propagation neural network (BPNN) controller [18], mean absolute value (MAV) and a support vector machine (SVM) [23], EMG-driven state space model which combines Hill-based muscle model with the forward dynamics of joint movement [22].

Hayashi et al. [19] reported a control method of robot suit HAL using biological information. The tests were conducted with a healthy subject, with two sensors near the flexor and extensor muscles during swinging motion of lower leg exercises. Signals were filtered and amplified, and the myoelectric activity was computed for both channels. Subsequently, the estimated muscle torque $\widehat{\mu }$ was calculated taking into account that

1 $$\begin{aligned} \widehat{\mu } = (a_{e}E_{e}(t)+b_{e}) - (a_{f}E_{f}(t)+b_{f}), \end{aligned}$$

where $E_{e}(t) \in \mathbb {R}$ and $E_{f}(t) \in \mathbb {R}$ are the myoelectric activity of the extensor and flexor muscles, respectively. Moreover, $a_{e} \in \mathbb {R}$, $a_{f} \in \mathbb {R}$, $b_{e} \in \mathbb {R}$ and $b_{f} \in \mathbb {R}$ are conversion coefficients from myoelectric activity to contraction torque. Finally, a gain parameter was used to compute the torque for the actuator. Their approach uses a simple algorithm, and it was tested online in a commercial robotic exoskeleton. However, their algorithm requires a long calibration process, including additional sensors such as torque sensors.

**Fig. 1:**
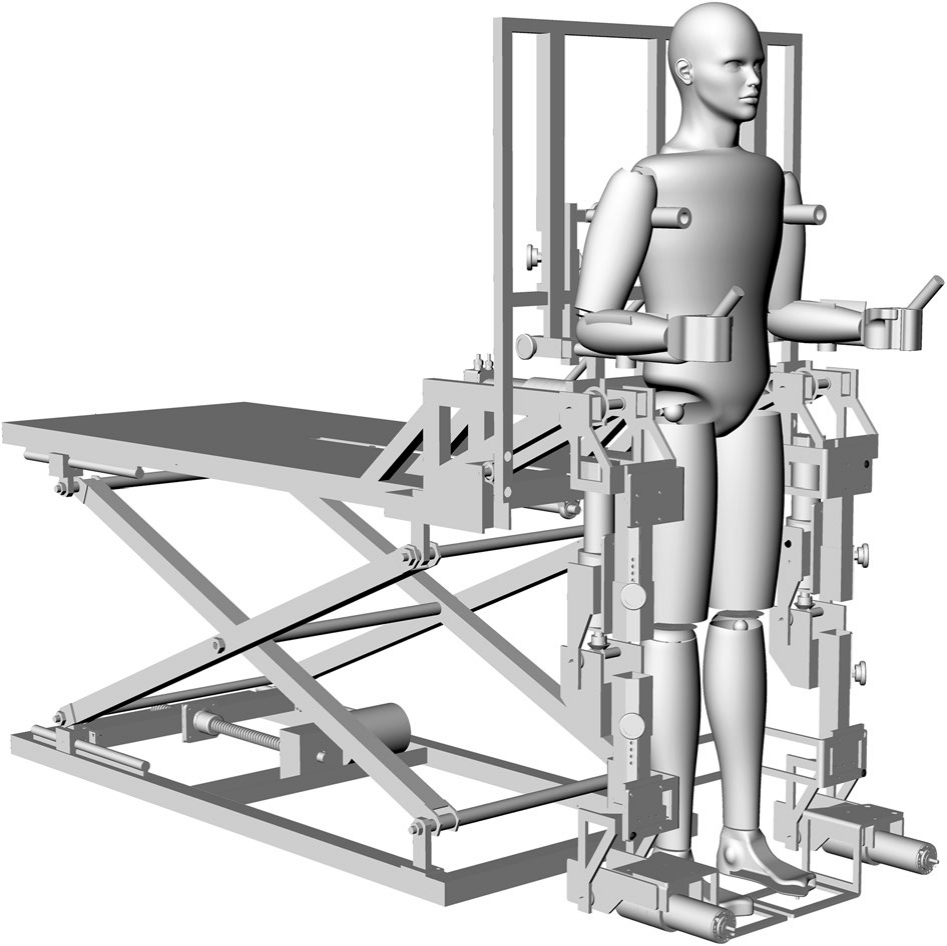
Nukawa, the robotic system for lower limb rehabilitation

The aim of the present study is to develop a myoelectric control (MEC) algorithm, based on the algorithm proposed by Hayashi et al. [19], but that does not require additional sensors and uses the maximum voluntary contraction (MVC) as a simple calibration process. The sEMG signal processing algorithm can detect the orientation and approximate the intensity of movement intention proportionally to the maximum MVC tests. The proposed MEC algorithm was implemented in a computational model of the lower limb rehabilitation system, Nukawa. Such a mechatronic system is a product of requirements presented by an interdisciplinary group, formed by physiotherapist and engineers, and has its antecedents in [25]. The mechanical design, presented in Fig. 1, consists of two limbs, each one composed by a three-link mechanism and a Computed Torque Control (CTC). The implementation of the CTC algorithm was conducted in a first stage as a hardware-in-the-loop (HIL), using the Nukawa simulation model without having to use the actual robot since Nukawa is not yet fully operational [26].

The three degrees of freedom allows each leg to perform flexion/extension (FE) movements of the hip, FE movements of the knee, and dorsi/plantar (DP) flexion movements of the ankle [25]. The design also has three brushless motors in each limb, power drivers, and encoders.

The joints are, approximately, collinear to human joints, and the system allows to adjust the length of each segment. The knee of the human body is a polycentric joint. However, a simplification was conducted, as presented by Zoss et al. [27], where a pure rotational joint in the sagittal plane was proposed for the exoskeleton. The system was designed for people from 1.44 to 1.85 m and up to 85 kg weight. The ROM of each joint was restricted with mechanicals stops considering the ROM for hip, knee, and ankle.

The MEC was conducted using a simulated model of Nukawa instead of the actual robot. Moreover, the sEMG signal processing algorithm and the movement control algorithm were implemented and tested with the simulated model, using an HIL simulation. The tests were conducted extracting signals from a sEMG signals collection, leading them into the real-time algorithms, and finally controlling the computational model of Nukawa.

The proposed MEC algorithm employs an estimated movement intention value of the knee joint. This estimation is mapped to the desired speed of the knee joint employing scaling factors. Such a speed is the input to the CTC algorithm of the simulated robotic system.

## Methods

This section presents the methodology used in the development of a sEMG signal processing algorithm to assess the detection of intended movement, based on the algorithm proposed by Hayashi et al. [19]. The algorithm was developed in both the offline programming environment MATLAB and as an HIL simulation in Python within a Beagle Bone Black (BBB) Rev C, which is a development platform.

### sEMG signal processing

This section proposes a sEMG signal processing algorithm, based on the algorithm stated by Hayashi et al. [19], to assess the detection of movement intention. The proposed sEMG signal processing algorithm can detect, approximately, the intensity of the motion intention proportionally facing the MVC. In this section, the signal processing algorithm was not conducted in real-time. However, tests were carried out with pre-recorded signals as proposed in the simulation-based methodology, stated by some of the authors in [28, 29].

**Fig. 2:**
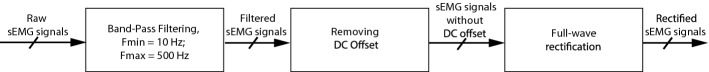
sEMG signal pre-processing subroutine

**Fig. 3:**
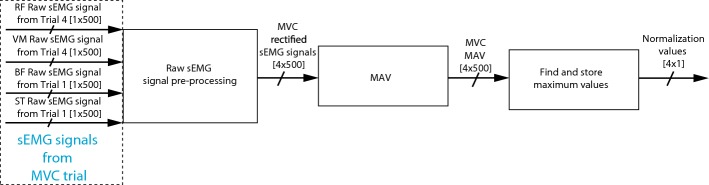
Subroutine to compute the four normalization values

**Fig. 4:**
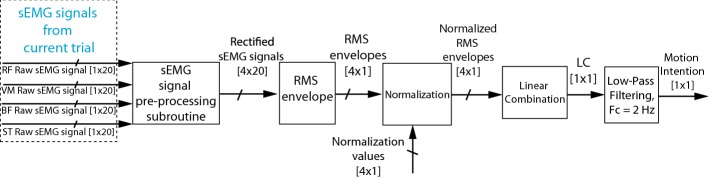
Main routine of the sEMG signal processing algorithm

Figures 2, 3 and 4 present the block diagrams that make up the sEMG signal processing algorithm. In these figures the notation [*n* × *m*] is the size of the signal bus, where *n* is the number of signals and *m* is the number of samples in the observation window.

Figure 2 presents a block diagram containing the principal functions of the sEMG signal pre-processing subroutine. In this figure it is possible to notice that the algorithm has three main blocks which are (1) band-pass filtering, (2) removing DC offset, and (3) full-wave rectification. This subroutine starts filtering the raw sEMG signals. A band-pass Butterworth filter with cut-off frequencies of 10 Hz and 500 Hz was used. A Notch filter was not used, since scientific recommendations from the SENIAM and the ISEK reports that EMG recordings should not use any notch filter [30, 31].

Besides, the mean of the sEMG signals is subtracted, to remove the DC offset. Subsequently, the subroutine performs full-wave rectification of the signals, computing the absolute value. The full-wave rectification process is conducted so that amplitude parameters such as the MAV or RMS can be applied to sEMG signals [32].

Figure 3 presents a block diagram containing the principal functions of the subroutine to compute four normalization values. In this figure it is possible to notice that the algorithm has three main blocks which are (1) raw sEMG signal pre-processing subroutine presented in Fig. 2, (2) MAV, and (3) finding and storing maximum values. The subroutine presented in Fig. 3 uses RF and VM signals, from Trial 4, and BF and ST signals, from Trial 1, to compute four normalization values, i.e., the MVC tests. These signals are later used to normalize the signals of these muscles, respectively. In the four cases, the algorithm extracts the MAV using adjacent windows of 500 ms, later the algorithm finds the maximum MAV, and it stores the maximum value obtained for each signal.

### Motion intention algorithm

Figure 4 presents a block diagram containing the principal functions of the main routine of the motion intention algorithm. In this figure it is possible to notice that the algorithm has five main blocks which are (1) sEMG signal pre-processing subroutine presented in Fig. 2, (2) RMS envelope, (3) normalization, (4) linear combination, and (5) low-pass filtering.

The main routine uses the four raw sEMG signals from the RF, VM, BF, and ST of current exercise and conducts them to the sEMG signal pre-processing subroutine. Subsequently, the algorithm extracts an RMS envelope of the four channels with sliding adjacent 20 ms windows since the algorithm should be fast and light, i.e., the total number of samples in a window from the vector of the signal was 20 samples. Afterward, the signals are normalized using the values previously stored for each of the channels during the MVC exercises, as previously mentioned in subroutine presented in Fig. 3. These signals are denoted as $RF_{RMS}$, $VM_{RMS}$, $BF_{RMS}$, and $ST_{RMS}$, which are the normalized RMS envelopes.

Finally, to detect the movement intention, a linear combination $LC \in \mathbb {R}$ of the four RMS envelopes is proposed, i.e., the features of four channels were combined. This *LC* is based on the algorithm proposed by Hayashi et al. [19], in which two channels were used. However, the conversion coefficients $a_{e}\in \mathbb {R}$, $a_{f}\in \mathbb {R}$, $b_{e}\in \mathbb {R}$, and $b_{f}\in \mathbb {R}$ are not estimated with an additional torque sensor, as proposed by Hayashi et al. [19], but determined heuristically. Moreover, the *LC* proposed in this paper uses four sEMG channels instead of two. To do so, the equation

2 $$\begin{aligned} LC = RF_{RMS} + VM_{RMS} - BF_{RMS} - ST_{RMS} \end{aligned}$$

was proposed, where $RF_{RMS}\in \mathbb {R}$, $VM_{RMS}\in \mathbb {R}$, $BF_{RMS}\in \mathbb {R}$, and $ST_{RMS}\in \mathbb {R}$ are the normalized RMS envelopes of the RF, VM, BF, and ST, respectively, taking into account that the RF and the VM muscles activate more during an extension intention. Moreover, the RMS envelope of these channels would be greater than the RMS envelope of the BF and the ST muscles during an extension intention. Therefore, the conversion coefficients of the $RF_{RMS}$ and the $VM_{RMS}$ have a positive sign, i.e., $a_{RF} = 1$, $b_{RF} = 0$, $a_{VM} = 1$, and $b_{VM} = 0$. The BF and the ST muscles activate more during a flexion intention. Therefore, the conversion coefficients of the $BF_{RMS}$ and the $ST_{RMS}$ are negative, since that these muscles are opposed to the RF and the VM muscles, i.e., $a_{BF} = -1$, $b_{BF} = 0$, $a_{ST} = -1$, and $b_{ST} = 0$. Therefore, when the subject intends to perform a knee flexion, the *LC* is negative in a comparable way to the MVC exercise for the flexion, and when the subject intends to carry out a knee extension, the *LC* is positive proportionally to the MVC exercise for the extension. Therefore, the motion intention of the proposed *LC* algorithm is a continuous value between − 2 and 2, i.e., $LC \in \left[ -2,2 \right]$, where − 2 and 2 are achieved during the MVC exercises in flexion and extension, respectively. Finally, the *LC* was filtered using a low-pass digital Butterworth Filter with a cut-off frequency of 2 Hz, order one, to remove the peaks and smooth the signal.

### Myoelectric control

This section shows how the motion intention algorithm presented before and the movement control algorithm, based on a Computed Torque Control (CTC) algorithm reported by the authors in a previous publication [26], were joined as an HIL simulation to create the MEC algorithm.

The protocol of the tests was carried out in real-time conducting the pre-recorded sEMG signals to the MEC algorithm. These signals correspond to those of the exercises mentioned in Table 1, specifically exercises 7–9, which correspond to concentric dynamic contraction of flexion and exercises 10–12 that correspond to concentric dynamic contraction of extension exercises. The tests assessed if the movement developed by the robotic system corresponds to the movement intention executed by the subject during the experimental protocol. Therefore, the tests did not involve individuals or animals but pre-recorded signals using a custom-made sEMG signal simulator.

**Table 1:** Exercises conducted during the experimental protocol

Exercise	Description
1	MVC (flexion) with the knee flexed at $90^{\circ }$ and the hip at $0^{\circ }$
2	75% isometric contraction (flexion) with the knee flexed at $90^{\circ }$ an the hip at $0^{\circ }$
3	50% isometric contraction (flexion) with the knee flexed at $90^{\circ }$ and the hip at $0^{\circ }$
4	MVC (extension) with the knee flexed at $90^{\circ }$ and the hip flexed at $90^{\circ }$
5	75% isometric contraction (extension) with the knee flexed $90^{\circ }$ and the hip flexed $90^{\circ }$
6	50% isometric contraction (extension) with the knee flexed at $90^{\circ }$ and the hip flexed at $90^{\circ }$
7	1RM (flexion) test with the knee flexed at $90^{\circ }$ and the hip at $0^{\circ }$
8	Concentric dynamic contraction (flexion) at 75% of the 1RM estimated in exercise 7, with the knee flexed at $90^{\circ }$ and the hip at $0^{\circ }$
9	Concentric dynamic contraction (flexion) at 50% of the 1RM estimated in exercise 7, with the knee flexed at $90^{\circ }$ and the hip at $0^{\circ }$
10	1RM (extension) test with the knee flexed at $90^{\circ }$ and the hip flexed $90^{\circ }$
11	Concentric dynamic contraction (extension) at 75% of the 1RM estimated in exercise 10, with the knee flexed at $90^{\circ }$ and the hip flexed at $90^{\circ }$
12	Concentric dynamic contraction (extension) at 50% of the 1RM estimated in exercise 10, with the knee flexed at $90^{\circ }$ and the hip flexed at $90^{\circ }$

A four component architecture was used to conduct the protocol of tests. The custom-made sEMG signal simulator is the first element. The simulator was developed in Python, a high-level programming language. The custom-made sEMG signal simulator extracts the signals from the computer and sends them from a computer to the BBB. The computer used for the tests was an $\hbox {Intel}^{\circledR }$ $\hbox {Core}^{\mathrm{TM}}$ i5 with a ${4\; \text {GB}}$ DD3 memory RAM. The computer communicates with the BBB through TCP/IP within a predefined communication port. The sampling period was set to ${\text {TS}} = 0.02\,{\text{s}}$. Therefore, the signals were extracted using a 20 ms window each time. The portion of the sEMG signals was conducted to the second component. A real-time implementation of the sEMG signal processing algorithm presented in Section Motion intention algorithm is the second component. The sEMG signal processing algorithm was implemented in real-time in a BBB which has an AM335x 1 GHz $\hbox {ARM}^{\circledR }$ ${\text {Cortex-A}8}$ processor and a 512 MB DDR3 Memory RAM. This implementation was also conducted using Python. The sEMG signal processing algorithm was developed in real-time as an HIL simulation, i.e., tests were performed using pre-recorded signals. Moreover, tests were carried out as proposed in the simulation-based methodology stated by the authors in [28, 29].

**Fig. 5:**
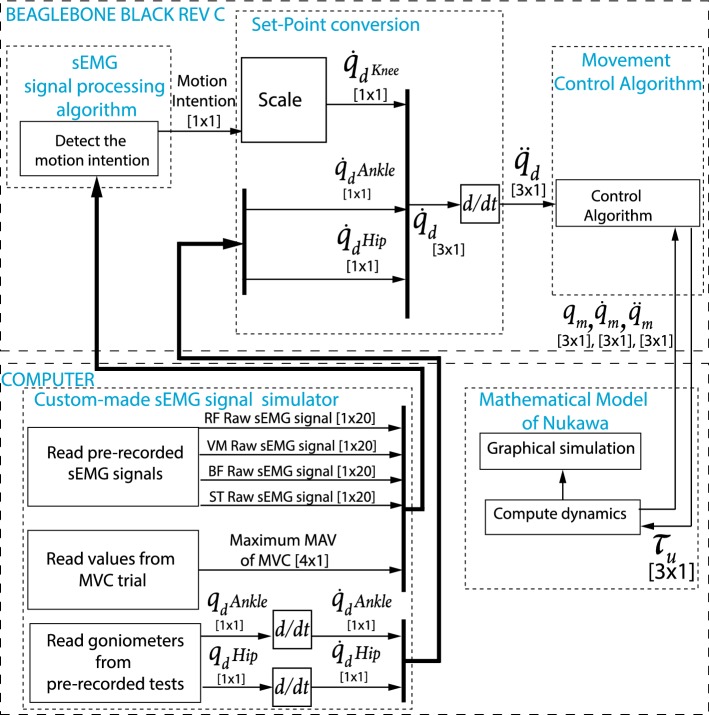
Set-point conversion for the MEC

The motion intention was sent through TCP/IP to the third component, which was the movement control algorithm presented by the authors in [26], and was also located in the BBB. To do so, a set-point conversion is conducted as shown in Fig. 5, i.e., the output of the motion intention algorithm *LC* is scaled taking into account that

3 $$\begin{aligned} \dot{q}_{d}\text {Knee} = \alpha LC +\beta , \end{aligned}$$

where $\dot{q}_{d}\text {Knee} \in \mathbb {R}$ is the desired speed for the knee joint, $\alpha \in \mathbb {R}$ is the amplitude scaling factor, and $\beta \in \mathbb {R}$ is the offset, two parameters left to the physiotherapist’s choice, according to the exercise. Subsequently, $q_{d}\text {Hip} \in \mathbb {R}$ and $q_{d}\text {Ankle} \in \mathbb {R}$ are derived, which are the desired angles given by the goniometers for hip and ankle joints, respectively. Therefore, $\dot{q}_{d}\text {Hip} \in \mathbb {R}$ and $\dot{q}_{d}\text {Ankle} \in \mathbb {R}$, the desired speed for the hip and ankle joints are obtained, respectively. In Fig. 5, the notation [*n* × *m*] is the size of the signal bus where *n* is the number of signals, and *m* is the number of samples in the observation window. The movement control algorithm is responsible for computing the torque $\tau _{u} \in \mathbb {R}^{3\times 1}$. The calculated torque $\tau _{u}$ is sent back to the computer through TCP/IP, to the fourth component, which is the mathematical model of Nukawa presented by the authors in [26].

The simulation of the dynamics of Nukawa is performed in the computer, in MATLAB, computing $q_{m} \in \mathbb {R}^{3\times 1}$, $\dot{q_{m}} \in \mathbb {R}^{3\times 1}$, and $\ddot{q_{m}} \in \mathbb {R}^{3\times 1}$ which represent the joint measured positions, velocities, and accelerations, respectively, i.e., after the simulation of the dynamics. Therefore, the graphic model moves as the desired path indicate it. Finally, an acknowledgment was sent back, and the loop was repeated each sampling period.

In order to validate that the MEC algorithm works correctly during actual exercises for rehabilitation of ACL injuries, six tests were conducted using the six dynamic exercises presented before, i.e., exercises 7–12. The graphic and numerical results of the six tests are shown below. These tests were carried out randomly, i.e., the combination of subject and exercise was randomized.

## Experiments and results

The tests of the algorithm were conducted in the offline programming environment MATLAB and as an HIL simulation in Python within a BBB Rev C. sEMG and kinematic signals of healthy subjects were obtained to test the algorithm. Finally, a test protocol was conducted to assess the behavior of the MEC algorithm for robot-assisted rehabilitation and its possibilities to aid rehabilitation therapies for ACL injuries.

### Subjects

An experimental protocol with 17 healthy subjects was conducted to record sEMG signals and its corresponding kinematics associated with rehabilitation body movements for ACL injuries. The ethics committee approved these tests.

Before each test all participants were deemed healthy under a clinical evaluation carried out by a health professional. Body weight, body height, blood pressure, heart rate, respiration rate, and body temperature were measured. Therefore, all of them were accepted in the study. The tests also recorded the age, suprapatellar perimeter, calf perimeter, inter-joint hip/knee distance, and inter-joint knee/ankle distance. The age of participants ranged from 19 to 47 years, with a median (interquartile range) of 25.5 years (23–30.5 years). Moreover, the body weight ranged from 50.1 to $81.9 \ \text {kg}$ and the body height ranged from 1.46 to $1.85 \ \text {m}$. In addition, the inter-joint hip/knee distance ranged from 0.35 to $0.44 \ \text {m}$ and the inter-joint knee/ankle distance ranged from 0.35 to $0.47 \ \text {m}$.

### Signal acquisition

In order to capture the movements performed by the subjects, the acquisition device was the wearable body sensing platform Biosignalsplux Professional (Plux, Lisbon, Portugal). The Biosignalsplux is a wireless device used to record and send real-time information captured by various sensors that can be connected. The sampling rate was configured to ${\text {fs}} = 1\, \hbox {kHz}$. The sensed data was stored using the OpenSignals software (Plux, Lisbon, Portugal). In order to capture the movements performed by the subjects during the selected experimental protocol, three twin axis goniometers (SG150) were used (Biometrics Ltd, Newport, UK). However, the tests only used the FE channels of each goniometer to measure hip FE movements, knee FE movements, and ankle DP flexion movements. The goniometers were located in the subject’s dominant lower limb. The location of the goniometers was conducted following some of the recommendations of the goniometer and torsiometer operating manual from Biometrics Ltd [33].

The sEMG sensor placement was determined based on some of the recommendations of the SENIAM Project [31]. According to the ISEK Standards for Reporting EMG Data [30] the characteristics of the procedure are shown:

The raw signal was detected using four pairs of commercial, disposable and adhesive gel surface electrodes placed in different parts of the upper leg of a group of healthy subjects, along with a reference electrode. The electrodes had a disc shape and were made of Ag/AgCl. They were placed with an interelectrode distance of approximately ${3.5 \; \text {cm}}$, center point to center point. The skin of fourteen subjects was shaved, and three subjects were not shaved. The area of interest was cleaned with alcohol before placing the electrodes to reduce the impedance between the electrodes and the skin. The electrodes were placed in order to detect flexion and extension of the knee, i.e., Rectus Femoris (RF), and Vastus Medialis (VM) muscles, detecting activation when the knee joint was extended, and Biceps Femoris (BF) and Semitendinosus (ST) muscles, detecting activation when the knee joint was flexed.

The electrodes were fixed parallel to the muscle fiber direction using the dominant middle portion of the muscle belly for best selectivity and avoiding the region of motor points. The signals were acquired using the Biosignalsplux. The device has a differential configuration, an input impedance of $100\,\hbox {G}\Omega$, CMRR of 100 dB, and it was configured with a gain of 1000. The biosignals were sampled at 1 kHz. The reference electrode was located on the Processus Spinosus of C7, in an electrically unaffected area.

To acquire the sEMG signals regarding ACL rehabilitation exercises, 12 exercises were conducted with each subject. Table 1 presents a description of the 12 exercises that were selected with the assistance of a physiotherapist with a graduate certificate in Biomedical Engineering. The test took approximately 2 h with each participant.

**Fig. 6:**
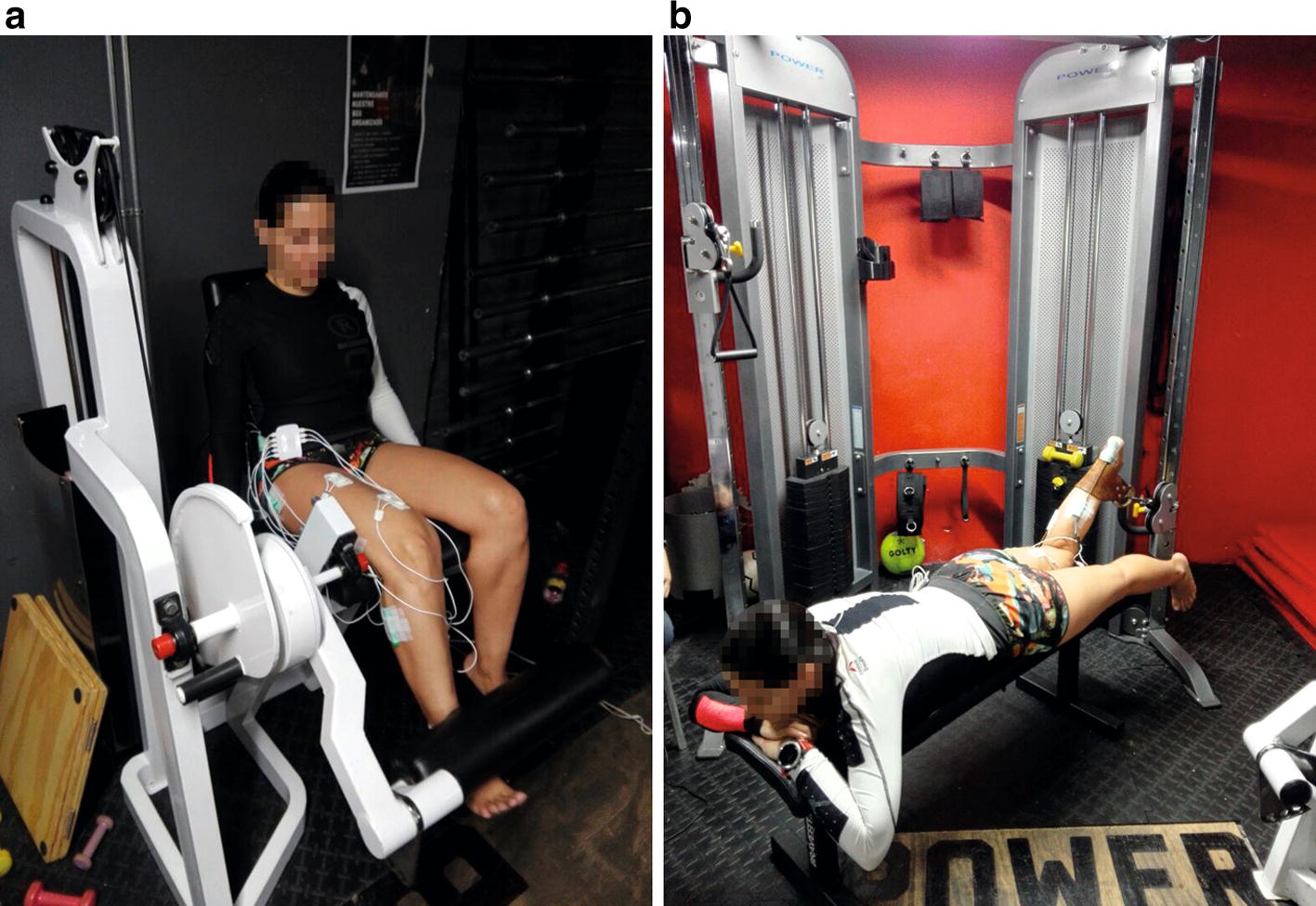
Gym machines used during the experimental protocol: **a** leg extension machine and **b** crossover machine

The physiotherapist selected six isometric exercises (1–6) and six concentric dynamic contraction exercises (7–12). Figure 6 presents two gym machines that were used during the experimental protocol for these two types of exercises. Figure 6a and b present the leg extension machine and the crossover machine, respectively.

The concentric dynamic contraction exercises were conducted taking into account the one-repetition-maximum (1RM) test. This test evaluates the maximum weight that an individual can lift only once for an exercise. Conducting the 1RM test may be contraindicated for some populations with preexisting medical conditions. Therefore, several 1RM strength prediction equations have been proposed, i.e., the 1RM can be predicted lifting the greatest weight possible for a certain number of repetitions, until fatigue [34, 35]. Some of the formulas were proposed by Lander [36], Brzycki [37], O’Connor et al. [38], and Epley [39]. Epley proposed that

4 $$\begin{aligned} \text {1RM} = w \left( 1 + \frac{r}{30}\right) = (0.0333 w )r + w \end{aligned}$$

where *w* represents the weight lifted by the subject and *r* is the number of repetitions executed, until fatigue. Equation (4) is widely employed due to its ease of use.

### Results of the offline implementation

**Fig. 7:**
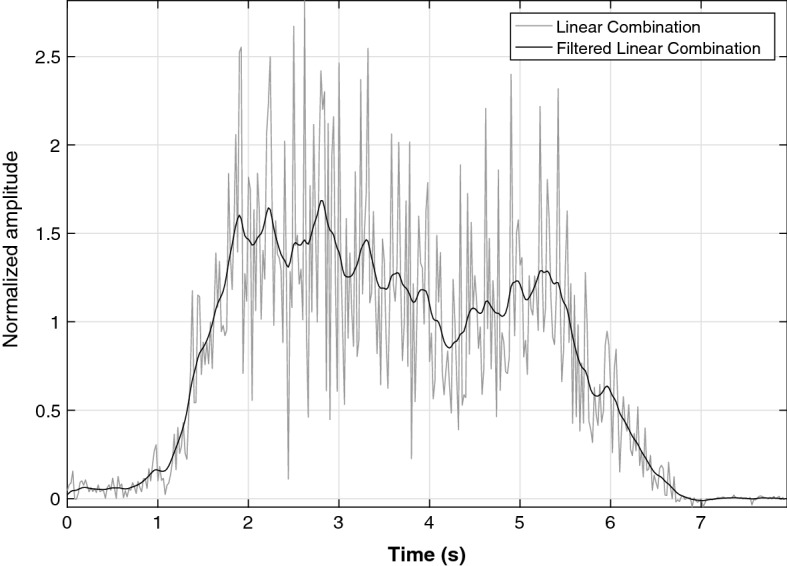
*LC* and *LC* filtered from exercise 4 of the S5

The tests of the sEMG signal processing algorithm were conducted with the signals acquired from the 17 healthy subjects. However, to exemplify the algorithm, the implementation with the signals obtained during the tests with the fifth subject (S5) is presented below (randomly selected). Figure 7 presents the results of the *LC* in light gray, and the *LC* filtered in black. In this figure, it can be observed the detection of the subject’s intention to perform an extension movement, since the *LC* filtered has a positive sign.

**Fig. 8:**
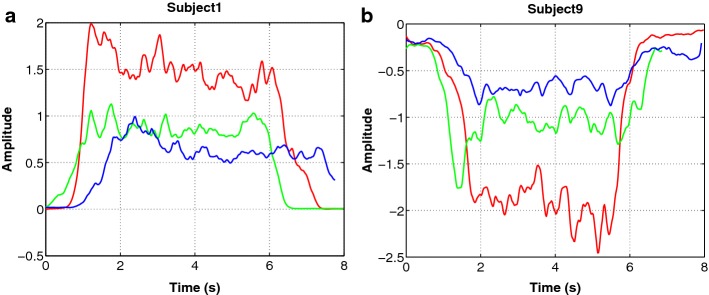
Linear combination from: **a** subject 1 during the three isometric extension exercises and **b** subject 9 during the three isometric flexion exercises. Red line represents the MVC extension exercise, green line represents the extension exercise with 50% of subjects body weight, and blue line with 75%

Figure 8a presents the results of conducting the signals of all three isometric extension exercises (4–6) from subject 1 to the motion intention algorithm. Figure 8b presents the results of conducting the signals of all three isometric flexion exercises (1–3) from subject 6 to the motion intention algorithm. Each subfigure has three lines, one for exercise. The red, green, and blue lines represent the detection of the motion intention *LC* for the MVC test, 75% isometric contraction, and 50% isometric contraction exercises, respectively.

### Graphic results of the protocol of tests

With the purpose of exemplifying the behavior of the MEC, the implementation with the signals obtained during exercise 9 with the seventh subject (S7) is presented below.

**Fig. 9:**
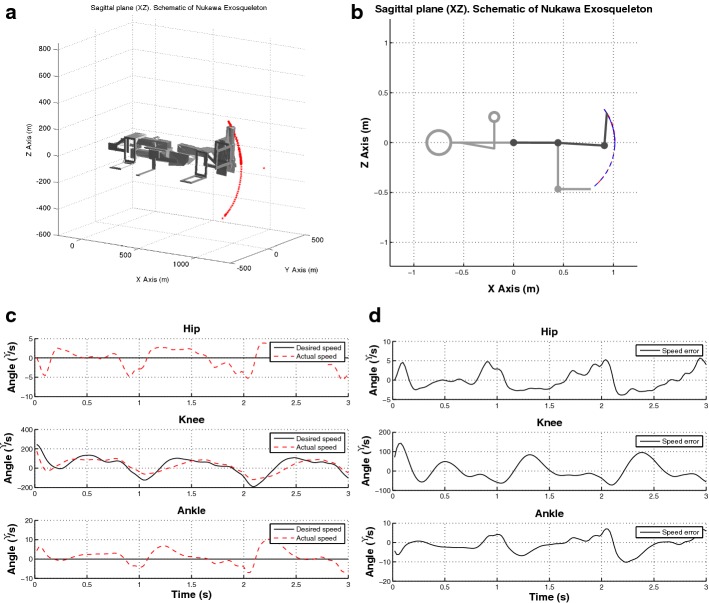
Results of the HIL simulation using a trajectory extracted during exercise 9 with S7 **a** 3D simulation, **b** simplified simulation, **c** desired speed vs. actual speed, and **d** speed error

Figure 9a–d presents the result of an HIL simulation for exercise 9 with S7. During exercise 9, the subject was prone on a flat bench with the knee flexed $90^{\circ }$, hip at $0^{\circ }$. Their ankle was fastened with a belt to a crossover machine. However, the simulations were conducted with the subject in a supine position, since Nukawa is not designed to perform therapies in a prone position. The above is acceptable for rehabilitation purposes since the exercises were selected taking into account international protocols for rehabilitation of ACL injuries, as presented in “Signal acquisition” section.

The online simulation presented in Fig. 9a was conducted using a 3D CAD model of Nukawa. This simulation included the kinematics of the robot. The simplified model of the robot was used as well, to reduce the computational time of the real-time tests. Figure 9b presents the result of the HIL simulation with the simplified model. In both figures, the red and dotted line represents the actual endpoint of the robot, i.e., the distal point of the third limb. In Fig. 9c presents the desired speed in a continuous line and the actual speed in a dotted line. In this figure, it is possible to observe that the system can follow the desired speed, i.e., the motion intention since both have similar behavior. Also, it can be denoted that the system follows the imposed set-point visualizing the error presented in Fig. 9d.

### Numerical results of the protocol of tests

The numerical results of the behavior of the MEC algorithm are shown in Table 2, which presents the trajectory tracking mean absolute error (MAE) of the control algorithm which was commanded with a set-point of $\dot{q}_{d}\text {Knee}$. As indicated in the table, the maximum position MAE is $0.1^{\circ }$, $6.3^{\circ }$, and $0.3^{\circ }$ for the hip, knee, and ankle joints, respectively. Thus, the error is lowest in the knee.

**Table 2:** Trajectory tracking error of the control algorithm which was commanded with a set-point of $\dot{q}_{d}\text {Knee}$

Subject	Exercise	$B_{r}$ ($^{\circ }$)	MAE position ($^{\circ }$, $^{\circ }$, $^{\circ }$)
7	9	180	(0.11, 5.953, 0.332)
11	10	90	(0.002, 1.209, 0.005)
14	8	180	(0.008, 2.270, 0.046)
18	12	90	(0.004, 2.031, 0.007)
20	11	90	(0.012, 6.355, 0.401)
12	7	180	(0.048, 4.997, 0.233)

For the above, the contribution of the MEC algorithm was validated for the implementation of robot-assisted rehabilitation of ACL injuries. During these therapies, the MEC algorithm would detect when the subject tries to move the knee, but due to the pain caused by the ACL injury, the patient is not able to execute the motion. Therefore, the MEC algorithm would assist its movement using the robotic system.

## Discussion

The novelty of the MEC algorithm proposed in this paper has two relevant characteristics. The first one is a simplified sEMG signal processing algorithm, to detect movement intention, that only requires an MVC test for calibration, i.e., it does not require additional sensors. The second one is that the motion intention was mapped to a speed set-point instead of a position or torque set-point, as is usually reported in the literature. A wider explanation of both characteristics is presented below.

To expand the information of the first characteristic, it is important to mention that some of the algorithms reported in the literature use a machine learning algorithm for the motion intention detection [17, 23, 40, 41]. Moreover, other algorithms use a model-based approach [22, 24, 42, 43]. However, those algorithms are more complex than the one reported in this paper. Therefore, they need more computing power. In the case of the proposed MEC algorithm, a simplified sEMG motion intention detection approach was achieved, similar to the ones proposed by [19–21]. The simplicity of the proposed algorithm makes it different from several approaches reported in the literature, where Artificial Neural Networks (ANN), Support Vector Machines (SVM), Hill-type muscular models, among others are used. This simplicity makes it easy to implement the algorithm in real-time. In comparison with other approaches that use machine learning algorithms, it is not necessary to perform high computational processes. A simple MVC calibration process is enough. The MVC test is used in most sEMG investigations to normalize the signals. Additionally, the proposed MEC algorithm requires no data sets, as the machine learning algorithms reported by other authors [40, 41, 44]. Since the sEMG signal is changing each session, it would be necessary to capture the MVC signal every time the algorithm is used, i.e., it requires an MVC exercise to obtain the calibration values for each session to process and detect the motion intention with EMG signals. Therefore, the MVC test may be conducted each time that the subject wears the robotic system to perform the simple calibration process. The information coming from sEMG signals was enough to detect the subject’s intention. No extra sensors, in addition to the sEMG electrodes, are required for the proposed MEC algorithm to work. Other approaches require additional sensors such as accelerometers, encoders, torque meter, goniometers, among others [17, 42, 45–47]. Additional sensors have the disadvantage that they deliver information about the intention after sEMG sensors and add extra costs. sEMG signals allow having an *a priori* estimation of the subject’s intention since sEMG signals appear before the muscle contraction is generated, i.e., the so-called electromechanical delay (EMD) [45].

The second characteristic is that the proposed MEC algorithm uses a velocity set-point. In this MEC algorithm, the motion intention was mapped to a speed set-point, using (3). Other algorithms [17, 18, 41, 43, 44] estimate the joint angle or even interaction torque, however, the proposed MEC algorithm detects the intention and orientation of the intention. This information is enough in the application for the robotic system Nukawa and can be useful for other areas such as biofeedback or interaction with robotic systems. Table 3 presents a comprehensive comparison with other sEMG motion intention algorithms. According to the results, an approximation of the intensity through a simplified algorithm was obtained despite not being the objective pursued. In this case, the intensity is unitless and is proportional to the MVC. The assumption is that, as reported in the state of the art if the coefficients of the *LC* were identified by the calibration process with additional torque sensors, the algorithm would estimate the torque.

**Table 3:** A comprehensive comparison with other sEMG motion intention algorithms

References	Robot	Sensors	Approximation	Implementation	Experiments	Calibration process	Type of Algorithm (black box, white box, grey box)	Results/errors	Advantages	Disadvantages
Proposed MEC algorithm	Nukawa	Four pairs of electrodes in BF, VM, RF, and ST muscles. One ground electrode in C7	A Butterworth filter with cut-off frequencies of 10 Hz and 500 Hz was used. Removing DC offset and full-wave rectification were also used. An *LC* of the four RMS envelopes is proposed, i.e., the features of the four channels were combined. The motion intention is a continuous value	Offline and online	17 healthy subjects	MVC exercise for both flexion and extension muscles	Grey box	The algorithm detected the orientation of the intention 100% of the times for both extension and flexion exercises. The algorithm detected the intensity of the movement intention, in a comparable way to the MVC, in 94% and 59% of the cases during extension and flexion exercises, respectively. The robot can follow the desired trajectories in Cartesian space, imposed by the MEC algorithm. The position and speed error are small compared to the motion of each joint. The maximum position MAE is $0.1^{\circ }$, $6.3^{\circ }$, and $0.3^{\circ }$ for the hip, knee, and ankle joints, respectively. Thus, the error is lower in the knee	It is a simple algorithm which requires a small amount of processor and no additional sensors. Moreover, the grey box model it uses a simple calibration process, using the well-known MVC tests. Also, it was tested on multiple subjects in comparison with other studies in its offline and online version	The *LC* proposed in this paper uses four sEMG channels instead of two
[17]	None	An accelerometer placed on the forearm and sEMG electrodes on BB and TB	The raw signal was processed. Subsequently, the signals were rectified, and a signal normalization process was developed for each subject according to pre-recorded signals. The neural activity was calculated, and a Kalman Filter was used to predict motion	Real-time	12 healthy subjects	Manual calibration	Black box	The model presented a high correlation with slow-motion trajectories (CC = 0.999–1). Moreover, the results showed high accuracy (97.4–98.6%) of prediction	It was validated with 12 healthy subjects and requires a simple manual calibration	It was tested on upper limb joints. Moreover, it requires additional sensors. The algorithm was tested in an offline fashion
[18]	A virtual human model (VHM)	The sEMG signals were collected from the anterior deltoid, posterior deltoid, BB, and TB	The signals were rectified with RMS to obtain an amplitude envelope. Subsequently, a low pass filter was implemented, and the signals were normalized. The raw and pre-processed signals were the input of a three-layer back propagation neural network (BPNN) controller	Offline	Four healthy subjects	Offline training of the algorithm	Black box	The ANN performance in the estimation of the joint angle in each motion was computed using the mean squared error (MSE) method. The worst average for the MSE was 0.239	It was tested using multiple joints and multiple movements	It requires to train a machine learning algorithm. Therefore, it requires a training data set, It was tested on upper limb joints. The algorithm was tested in an offline fashion
[19]	HAL-3	Two sensors near the flexor and extensor muscles	Signals were filtered and amplified, and the myoelectric activity was computed for both channels. Subsequently, the estimated muscle torque was computed as a linear combination of both, taking into account the equation of a straight line. Finally, a gain parameter was used to compute the torque for the actuator	Online	A healthy subject	A calibration process is necessary to obtain the conversion coefficients	Black box	The conversion coefficients depend on the sensor location and the operator’s physical condition	It uses a simple algorithm, and it was tested online in a commercial robotic exoskeleton	It requires a long calibration process, including additional sensors such as torque sensors
[20]	A computer model of the index finger and wrist joints	Flexor digitorum superficialis (FDS) and flexor carpi ulnaris (FCU)	An RMS envelope was computed. Subsequently, a low-pass filter was used, and two different functions were used for the finger position	Online	18 healthy subject	Simple calibration process of constants	Black box	The maximum errors obtained were $3.77^{\circ }$. A direct relationship between the RMS and the motion of model was observed	It uses a simple algorithm, and it was tested in an online fashion. It requires a simple calibration process, and it was tested on 18 healthy subjects	It was tested on upper limb joints
[21]	NEURO-exos	BB and TB	The EMG signals were processed obtaining a linear envelope (LE) through full-wave rectification. Both signals were conducted to a proportional controller to manipulate the flexion and extension of the exoskeleton	Online	Ten healthy subjects	Subjects selected the gains of the algorithm in a previous procedure	Black box	Subjects could fulfill the tasks during all trials, no matter the percentage of assistance, extra weight or movement pace	It requires a simple calibration exercise, it was tested in an online fashion, and it was tested on ten healthy subjects	It was tested on upper limb joints
[23]	None	BB and TB	The MAV was computed. Subsequently, discriminant analysis and an SVM was used to classify the signals	Not reported	Three healthy subjects	Training of the algorithm	Black box	Classification accuracy for the discriminant analysis and the SVM was 96% and 99%, respectively	It was tested using multiple joints and multiple movements	It requires to train a machine learning algorithm. Therefore, it requires a training data set, it was tested on upper limb joints. The type of implementation is not reported
[24]	None	BB and TB	A low-pass filter was used. Subsequently, two time-domain features were extracted and the signals were normalized. A linear state-space model was used to estimate joint motion	Offline	Two healthy subjects at two load levels	Offline training of the algorithm	Black box	The authors obtained a root-mean-square error ranging between 8.3 and 10.6%. Also, the prediction error of the average angle was around 10%	It overcomes subject-specific problems	It was tested in an offline fashion. It was tested on upper limb joints and just in two subjects
[41]	iLeg	RF, VL, VM, BF and ST	Full wave rectification, low pass filter of 2 Hz of the sEMG signals which are inputs of a network Neural network. The angle and speed are also inputs to the neural network	Offline	One healthy subject	Training the neural network	Black box	The root-mean-square error is 0.67 N m for hip torque estimation and 0.37 N m for knee torque estimation	It can be used to perform a real-time coordinated active training with a rehabilitation robot. It was tested using multiple joints, hip, and knee	The proposed approach was tested with a circular-like trajectory. Requires additional sensors to measure angular position and speed
[43]	Actuated leg-orthosis system	VL, RF and ST muscles	The sEMG signals were full wave rectified. Subsequently, a low pass filter was used. Finally, the processed signals were used as inputs of a Hill-type muscle model	Online	One healthy subject	The experimental torque was computed by employing the inverse dynamics	White box	From the experimental results the authors obtained a calibration accuracy with an RMSE ranging between 1.49 and 1.99 N m and the average R2 was 0.89	The calibration process is subject-specific. The algorithm uses a white-box model, which makes it easier to understand. It was tested in an online fashion	The model requires knowing parameters such as the lengths of the muscles involved. The study only considers the knee joint
[44]	None	Muscles of the quadriceps	Adaptive neural networks and fuzzy logic	Offline	One Healthy subject	Training the neural network and set the inference rules of the fuzzy logic	Black box	The performance of the algorithm after least square reached the desired torque level with a mean square error of 181.8	This model uses different types of EMG-Torque profiles in one neural network. Many muscle activation profiles are used to estimate knee joint torque at different impedance levels that experiment the patient	It requires a training data set. Furthermore, it needs to set inference rules for the fuzzy logic
[40]	None	VL	RMS of the sEMG signal. Subsequently, a particle swarm optimization (PSO) technique was used	Offline	One healthy subject	Training the algorithm	Black box	A Torque sum squared error ranging between 6148.26 and 25330.10. An average coefficient of determination $(R^{2})$ of 0.88	The mathematical model for torque estimation is easy to implement since the equations are simple	The algorithm was tested on a single muscle and a single joint (knee)
[42]	None	sEMG signals collected from VM, VL, Vastus intermedius and RF. Knee angle	The sEMG signals were rectified, a 6 Hz low pass filter was used, and the signals were normalized to be used as inputs to a Hill-type muscle model. The parameters of the model were optimized with quadratic minimums from a nominal torque signal and the torque signal estimated by the model	Offline	One healthy subject	Training the torque estimation algorithm	White box	The lowest error corresponds to the Sequence iii proposed by the authors and the cost function 1, also proposed by them 0.68%	The torque found after the identification of muscle parameters tendon can be used to detect the parameters of a model with reasonable accuracy	For the torque estimation, they used additional sensors such as an isokinetic dynamometer to measure torque and angular position. The latter is input to the muscle model
[48]	HAL 3: four-link and three-joint	Extensor and the flexor of the knee and the hip	Method to assist motion through torque assistance corresponding to the operator’s intention	Online	One healthy subject	Manual calibration	Black box	With an assist ratio $\hbox {Gr} = 0.6$, the result shows that EMG and the assist torque approach the constant values during walking. This result means that the myoelectricity is controlled by adjusting the assist torque	The algorithm is designed to assist movement and torque when walking	The algorithm was tested on a single healthy subject and uses additional floor reaction force sensors
[45]	Leg exoskeleton	sEMG signals collected from RF, VL, and ST. Force and hall sensors	A dynamic human body model and the DFC of the actuator. In both approaches, a high-level control loop evaluates EMG signals and the current state of the human body and orthosis. The output is the desired motion expressed, as either the desired knee angle or torque	Online	One healthy subject	Isometric contractions of the knee flexor and extensor muscles without floor contact for the RF and ST are used for calibration	White box	The knee torque derived from the EMG signals is significantly lower compared to the trial without support	The algorithm used a function to obtain the force from the signal of sEMG to be used as input in the biomechanical model and thus be able to obtain the torque of the knee	The algorithm was designed to work only in the knee joint and was tested only to climb a step with two levels. The algorithm only was tested with a healthy subject
[49]	KAFO	Left soleus (Sol), tibialis anterior (TA), VL and medial hamstrings (MH)	A physiologically-inspired controller to control artificial muscle forces using sEMG signals. Each artificial pneumatic muscle is controlled by a sEMG signal generated by a biological muscle, e.g, at the knee, they used VL to control the two artificial knee extensors and MH to control the two artificial knee flexors	Online	Three healthy male subjects	Simple tests were carried out to verify that the electrodes placement give appropriate signals for each muscle	White box	This robot produced a 22–33% of the peak knee flexor moment, a 15–33% of the peak extensor moment, a 42–46% of the peak plantar flexor moment, and a 83–129% of the peak dorsiflexor moment, all of this during regular walking	The algorithm includes an inspired control of the physiology of the knee and ankle. This algorithm controls every artificial pneumatic muscle with EMG signals	The algorithm was only tested in 3 healthy subjects. An additional component is needed to manage the pneumatic muscles
[46]	Exoskeleton with 2-DOF, hip and knee	EMG signals from the biceps muscle and quadriceps muscle of the thigh. Also, the angle and the interaction forces are measured	They proposed a bidirectional human–machine interface including a neuro-fuzzy controller, based on EMG signals, and extended physiological proprioception (EPP) feedback system is developed by imitating the biological closed-loop control system of the human body	Online	A healthy male subject and a healthy male subject	sEMG signals and interaction forces were used to train the neuro-fuzzy network	Black box	The interaction force of the controller without the EMG feeding-forward item is more significant. The average value is 22.65 N after 100 tests, while the average value of the controller with EMG feeding-forward item is 12.46 N, which is 44.97% smaller than the previous one	The algorithm includes an extended physiological proprioception feedback system. It uses a neuro-fuzzy controller to decode human movement using sEMG signals that reflect the intention of the movement and the proprioception of angular feedback	The algorithm was only tested with two subjects. The system needs data previous to be used as a training sample to modify the parameters in the neuro-fuzzy network
[47]	Exoskeleton system with 1-DOF joints: hip and knee	Information including sEMG, joint angle, and force are collected and analyzed in real time	Active-compliance control of the human–machine system is established based on real-time muscle force estimation and human–machine interactive force detection, while progressive treatment in accordance with stroke stage is realized by timely evaluation. EPP feedback system based on tactile stimuli is developed to help rebuild the closed-loop control system of the human body	Online	Three healthy male subjects	Not reported	White box	During the FE exercises, the interactive force remains from − 10 N to 10 N and the RMS value is 4.35 N, it indicates the exoskeleton joint can follow the movement of the human knee	In their rehabilitation system, an active coupling is mounted on a standing bed. It is designed to guarantee a comfortable and safe rehabilitation according to the structure and control requirements	The algorithm was tested on three healthy people. Besides, it uses an additional sensor for the extended physiological proprioception system, which generates greater data processing

Some limitations of this study are: The proposed algorithm was tested on both offline and online. However, the results cannot be generalized to the entire population, only to the sample, i.e., the study population is not statistically significant to generalize the results. Also, as the tests were performed on healthy subjects, it is still not possible to conclude about the behavior of the MEC algorithm with sEMG signals from subjects with ACL injuries. Therefore, the results obtained cannot be extrapolated directly to people with this type of injury. This restriction also applies to all approaches reported in the literature that conducted the tests with healthy subjects where the extension to other conditions must be proven. Also, the experimental protocol did not consider to measure or control the factors that affect the sEMG signals, e.g., the environmental temperature, the body temperature, the skin impedance and location of the electrodes. Therefore, it is not feasible to conclude if the proposed MEC algorithm is affected by these factors.

Finally, CTC is a model-based control which enables compliant robot control with small tracking errors for accurate robot models. Nevertheless, the proposed MEC algorithm was tested only with this controller. Therefore, future work includes several tests to the MEC algorithm with other control algorithms to assess the robustness.

## Conclusions

Surface electromyography (sEMG) signal processing algorithm, based on the algorithm reported by Hayashi et al. in [19], was proposed. The proposed algorithm detects the motion intention in the knee joint and requires no prior training with sEMG signals from other subjects. Moreover, no additional torque sensor is required to estimate the conversion coefficients from the Linear Combination (*LC*) algorithm.

The results showed that when a subject intended to perform a knee flexion or extension, without executing the movement, the algorithm detected the orientation of the movement intention. Moreover, when a subject intended to carry out an extension movement, the algorithm detected an *LC* with a positive sign, and when a subject intended to perform a flexion movement, the algorithm detected an *LC* with a negative sign.

The behavior of the myoelectric control (MEC) algorithm for robot-assisted rehabilitation and its possibilities to support rehabilitation therapies for ACL injuries was tested through a protocol of tests. Both algorithms were joined together, i.e., the sEMG signal processing algorithm, and the movement control algorithm. The protocol of tests was conducted as an HIL simulation conducting the pre-recorded sEMG signals to the MEC algorithm. The results of the HIL simulations shown that the MEC algorithm is a potentially useful tool for the implementation of a robot-assisted rehabilitation protocol for ACL injuries. However, this proposal cannot be generalized for the entire population, but can only be considered for the sample, i.e., the 17 healthy subjects of the people who participated in the study.

The main contribution of this paper is the combination of two algorithms to propose a MEC algorithm. The arrangement reveals something useful to perform robot-assisted therapy for ACL injuries. The algorithm detects the motion intention and controls a robotic rehabilitation system to assist the knee movement, i.e., such as in active-assisted extension exercises but with an exoskeleton.

In conclusion, the proposed MEC algorithm improves upon previous alternatives since it is a simple algorithm which requires a small amount of processor and no additional sensors. Future work includes several tests with pre-recorded signals and the actual robot, i.e., to test the MEC algorithm with the real robot and pre-recorded signals. Also, it is possible to extend the endorsement from the ethics committee to conduct several tests with healthy subjects with the Biosignalsplux (Plux, Lisbon, Portugal) or any commercial acquisition device, or even to perform a clinical Trial to assess the behavior of the MEC algorithm but with patients, not just with healthy subjects. Finally, future work includes to test and evaluate the MEC during a rehabilitation process with Nukawa.
